# The Effect of Teriparatide Treatment on the Risk of Fragility Fractures in Postmenopausal Women with Osteoporosis: Results from the Asian and Latin America Fracture Observational Study (ALAFOS)

**DOI:** 10.1007/s00223-021-00895-4

**Published:** 2021-08-20

**Authors:** Chung-Hwan Chen, Abdulaziz H. Elsalmawy, Sophia Ish-Shalom, Seung-Jae Lim, Nadia S. AlAli, Joao L. Cunha-Borges, Huilin Yang, Noemi Casas, Lale Altan, Zhanna Belaya, Fernando Marin, Thomas Moll, Sirel Gurbuz, Alan Brnabic, Imre Pavo, Sandra Florez

**Affiliations:** 1 Department of Orthopedics and Orthopedic Research Center, Kaohsiung Municipal Ta-Tung Hospital and Kaohsiung Medical University Hospital, College of Medicine, Kaohsiung Medical University, Kaohsiung, Taiwan; 2 Regeneration Medicine and Cell Therapy Research Center, Kaohsiung Medical University, Kaohsiung, Taiwan; 3 Department of Trauma and Orthopedic Surgery, Al Noor Specialized Hospital Makkah, Mecca, Saudi Arabia; 4 Lin Endocrine Research Center, Haifa, Israel; 5 Department of Orthopedic Surgery, Samsung Medical Center, Sungkyunkwan University School of Medicine, Seoul, South Korea; 6 Endocrinology Unit, Amiri Hospital, MOH, Kuwait City, Kuwait; 7 Centro de Pesquisa Clinica do Brasil, Brasília, Brazil; 8 Department of Orthopedics, The First Affiliated Hospital of Soochow University, Suzhou, People’s Republic of China; 9 Riesgo de Fractura CAYRE, Bogotá, Colombia; 10 Department of Physical Medicine and Rehabilitation, Uludağ University School of Medicine, Bursa, Turkey; 11 Department of Neuroendocrinology and Bone Disease, The National Medical Research Center for Endocrinology, Moscow, Russia; 12 Eli Lilly and Company, Indianapolis, USA; 13 Pain and Palliative Care Department, Universidad de la Sabana, Chía, Colombia

**Keywords:** Fracture, Observational study, Osteoporosis, Teriparatide, Back pain

## Abstract

The Asian and Latin America Fracture Observational Study (ALAFOS) is a prospective, observational, single-arm study conducted in 20 countries across Asia, Latin America and the Middle East. ALAFOS evaluated new clinical vertebral and non-vertebral fragility fractures in relation to time on teriparatide, in postmenopausal women with osteoporosis in real-life clinical practice. Clinical fragility fractures, back pain, and health-related quality of life (HRQoL) were recorded in 6-month intervals for ≤ 24 months during teriparatide treatment and up to 12-months post-treatment. Data were analysed with piecewise exponential regression with inverse probability weighting for time to event outcomes and mixed-model repeated measures for back pain and HRQoL. 3054 postmenopausal women started teriparatide and attended ≥ one follow-up visit (mean [SD] age 72.5 [10.4] years). The median (95% CI) time to treatment discontinuation was 22.0 months (21.2, 22.8). During the treatment period, 111 patients (3.6%) sustained 126 clinical fractures (2.98 fractures/100 patient-years). Rates of new clinical fragility fractures were significantly decreased during the > 6–12, > 12–18, and > 18–24-month periods, as compared with the first 6 months of treatment (hazard ratio [HR] 0.57; 95% CI 0.37, 0.88; *p* = 0.012; HR 0.35; 95% CI 0.19, 0.62; *p* < 0.001; HR 0.43; 95% CI 0.23, 0.83; *p* = 0.011; respectively). Patients also reported an improvement in back pain and HRQoL (*p* < 0.001). These results provide data on the real-world effectiveness of teriparatide in the ALAFOS regions and are consistent with other studies showing reduction of fractures after 6 months of teriparatide treatment. These results should be interpreted in the context of the noncontrolled design of this observational study.

**Supplementary Information:**

The online version contains supplementary material available at 10.1007/s00223-021-00895-4.

## Introduction

Osteoporotic fractures lead to acute pain, increased morbidity and mortality and ultimately a lower quality of life as well as higher health care costs [1]. It is estimated that by 2040, the number of patients with a high risk of osteoporotic fracture will reach over 300 million worldwide, presenting a significant disease burden to society [2].

In 2000, there was an estimated nine million new osteoporotic fractures worldwide [3], and as the number of people 65 years and older increases, the number of hip fractures alone is expected to reach 6.26 million globally by 2050 [4]. While the frequency of osteoporotic fractures (including hip, vertebral, and non-vertebral) varies widely globally, osteoporosis presents a growing burden in Asia, Latin America, and the Middle East [5–7]. Cooper et al*.,* predicted that, by 2050, Asia will account for approximately 51% of global hip fractures in women aged 65 and over. Moreover, Asia, Latin America, and the Middle East together will account for almost 70% of global hip fractures in women aged 65 and over [4]. The prevalence of vertebral fractures varies widely, with the rates of vertebral fractures in women aged over 50 years, ranging from 11 to 19%, in Latin American countries, 5% to 30% in Asian countries, and 20% to 46% in Middle Eastern countries [8].

Treatment for osteoporosis focuses on inhibiting bone resorption and/or increasing bone formation. Bisphosphonates, denosumab, estrogen-receptor modulators and estrogens all reduce bone resorption, while romosozumab decreases bone resorption and increases bone formation. Teriparatide, the first approved drug to increase bone formation, is a recombinant human parathyroid hormone ([PTH] 1–34) that increases bone mass and quality by stimulating osteoblast activities [9, 10]. Teriparatide improves bone microstructure by stimulating trabecular and cortical bone formation, thereby reversing osteoporotic bone deterioration [11].

Randomised controlled clinical trials (RCTs) have established the efficacy and safety of teriparatide and have shown that patients receiving teriparatide have a decreased risk of fracture as compared to those on placebo, alendronate, or risedronate [9, 12, 13]. Of note, results from the Fracture Prevention Trial suggest that longer duration of teriparatide treatment is associated with a reduction in non-vertebral fracture rate and a decrease in back pain [14, 15].

In line with the RCTs, several observational studies have demonstrated the effectiveness of teriparatide in the United States (USA), Europe, and Japan [16, 17]. The Direct Assessment of Non-vertebral Fractures in Community Experience (DANCE) study in the USA reported a 43% decrease in the incidence of non-vertebral fractures during the last 6-month period of teriparatide treatment (18–24 months), as compared to the first 6-month period, and this reduction was maintained during the post-treatment 24-month follow-up [18]. Similarly, the European Forsteo Observational Study (EFOS) and the Extended Forsteo Observational Study (ExFOS) reported a 39% and 47% decrease in the odds of clinical fractures respectively, for patients in the 12 to 18-month period as compared to the first 6-month period [19, 20]. In addition, the Japan Fracture Observational Study (JFOS) reported a 59% reduction in the clinical fractures risk in the last 6-month period of teriparatide treatment (18–24 months) as compared to the first 6-month period in Japanese patients [21]. Furthermore, integrated analysis of these four studies reported a decrease in the rate of hip fractures during the > 6-month period as compared with the first 6-month period [16, 17]. All four observational studies reported a reduction in back pain and an increase in quality of life for patients treated with teriparatide [18–22].

These studies show associations between teriparatide treatment and a lower risk of fractures and a decrease in back pain in the USA, Europe, and Japan. However, large-scale evidence of teriparatide’s effectiveness as well as treatment persistence and adherence data in real-life clinical settings in Asia, Latin America and the Middle East is lacking. There are a variety of differences between these geographies and the previously studied populations in the USA and Europe. In the Asian and Latin America Fracture Observational Study (ALAFOS) participant countries, there is a rapidly aging population, an increasing incidence of osteoporosis, a high proportion of patients who are treatment naïve, and variations in clinical practices and guidelines for the treatment of osteoporosis [4, 23–26]. Additionally, Asia–Pacific and South American populations have a lower calcium intake as compared to Western populations. Many countries in South, East, and Southeast Asia intake < 400 mg of calcium a day, while countries in South America have moderately low calcium intake (between 400 and 700 mg/day) [27]. Similarly, an inadequate level of vitamin D is common in Asia, Latin America, and the Middle East [5–7].

This gap in knowledge on the effectiveness of teriparatide in Asia, Latin America, and the Middle East, combined with the fact that the burden of osteoporotic fractures is predicted to increase in these regions over the next 30 years [28] highlights the necessity of investigating the effectiveness of osteoporosis drug treatments in a real-world setting in these geographies.

We report here the analysis of the association between rates of patients with incident clinical vertebral and non-vertebral fragility fractures and duration of teriparatide therapy as part of routine clinical practice, treatment adherence, and HRQoL in postmenopausal women enrolled in the ALAFOS study.

## Materials and Methods

### Study Design and Participants

ALAFOS is a prospective, observational, outpatient, single-arm study. The study design and patient baseline characteristics have been previously described [25]. In summary, 3098 postmenopausal women with osteoporosis were enrolled between December 2015 and October 2017, from 20 countries across the three regions. The study consisted of two phases: an active treatment phase in which patients received teriparatide for up to 24 months and a follow-up phase in which patients were followed for up to 12 months after teriparatide treatment. Eligible patients were postmenopausal women at a high risk of fracture, who were teriparatide and PTH 1–84 naïve, and started teriparatide treatment (20 μg/day subcutaneous injection) as part of routine clinical practice between 2 weeks before or 4 weeks after entry into the study. Patients were excluded if they were currently being treated with an investigational drug or procedure or had any contraindications as described on the teriparatide label [25]. A sample size of 3000 was determined based on the primary outcome. Full details are in the supplementary materials. Before enrolment, all patients gave written informed consent and were able to withdraw from the study without consequence at any time. The study was approved by local ethics committees or review boards, depending on local requirements.

### Data Collection

Patients were observed within the normal course of clinical care, for up to 36 months following study entry. Data were collected at the baseline visit, at approximately 3, 6, 12, 18 and 24 months after starting treatment, and at approximately 6 and 12 months after discontinuing treatment. Data collected at baseline included patient demographics, clinical risk factors, and socioeconomic factors. Full details of baseline data collection has been previously described [25].

#### Medication Persistence and Adherence

At each follow-up visit during the teriparatide-treatment phase, patients were asked whether treatment was interrupted for more than 4 consecutive weeks (28 days) since the previous visit. Patients were asked to provide the estimated start and stop dates for each interruption and estimate the number of injections they missed, including any treatment interruptions with a duration of less than 4 consecutive weeks. The reason for stopping study treatment was also collected for all patients.

Teriparatide adherence was assessed using the medical possession ratio (MPR), the sum of daily teriparatide injections between the reported start and stop dates divided by the total number of days in that period adjusted for any patient-reported treatment interruptions and missed injections. For this study, MPR ≥ 80% of the observation period time was considered as high adherence and MPR < 50% was considered as low adherence.

#### Fractures

The primary endpoint was time to first new clinical fragility fracture, either vertebral or nonvertebral, measured in days. Incident clinical vertebral and non-vertebral fragility fractures, were recorded at each visit after initiating teriparatide treatment. If the patient had a new fracture, the anatomical location and date as well as the level of trauma that caused the fracture were recorded. Fracture validation was based on medical confirmation by X-ray, imaging techniques, emergency room report, surgical report, or physician’s confirmation. Non-vertebral (clavicle, scapula, ribs, sacrum, humerus, radius, ulna, carpus, pelvis, femur, patella, tibia, fibula, ankle calcaneus, tarsus, metatarsal, hip, femoral diaphysis, and distal femur) and vertebral fractures at T4 through L4 were recorded.

#### Health-Related Quality of Life

The HRQoL was self-assessed using the 5-level EuroQol-5 dimension (EQ-5D-5L) questionnaire. Patients self-assessed at baseline, at approximately 12 and 24 months after starting teriparatide treatment, and 12 months after treatment discontinuation. The EQ-5D-5L comprises five dimensions of health: mobility, self-care, usual activities, pain/discomfort, and anxiety/depression; and each dimension comprises five levels: no problems, slight problems, moderate problems, severe problems, and extreme problems [29]. The United Kingdom scoring algorithm was used to calculate a single score from the domain scores.

#### Back Pain

Back pain severity was evaluated using the back pain numeric rating scale (NRS) [30]. During routine observations patients were asked to rate the worst and average pain experienced in the previous 24 h on a scale of 0 (no back pain) to 10 (worst possible back pain). Patients were asked to complete the questionnaire at baseline, at 6, 12, 18 and 24 months after treatment initiation, and12 months after treatment cessation.

#### Safety

Protocol defined adverse events (AEs) to be collected throughout the study were all fatalities in temporal association with teriparatide, all fractures and any fracture-related hospitalisations or fracture-related surgeries, and AEs or serious AEs (SAEs) that led to the discontinuation of teriparatide treatment [25].

### Statistical Analysis

All patients with baseline data who received at least one dose of teriparatide and returned for at least one post baseline visit were included in the analysis. All models and analyses of the primary objectives were prespecified in a statistical analysis plan. Baseline characteristics were summarised using descriptive statistics (mean, standard deviation [SD] or median with interquartile ranges [Q1, Q3]). Kaplan–Meier estimates were used to calculate time to treatment persistence. This used the date from the start of treatment to the discontinuation of treatment. Death, withdrawal from study, and the end of the study period were censored. All patients without any gaps in treatment of more than 90 days were considered ‘persistent’. Treatment adherence was measured by MPR and was dichotomised at greater than or equal to 80% versus less than 80%.

Piecewise exponential regression (PER) analyses with inverse probability of treatment weighting (IPTW) were used to assess the time to first clinical fragility fracture over 6-month intervals. This approach accounts for both time to fracture and censors patients who do not experience the event, are lost to follow-up, left the study or died. The inverse probability weights were constructed as 1 for patients using teriparatide for 0 to 6 months and 1/(the predicted probability of use of teriparatide for 6–24 months) for patients using teriparatide for 6 to 24 months. Propensity scores used for the IPTW were calculated from a logistic regression model with use of teriparatide as the outcome and the following covariates; baseline comorbidity (yes/no), history of fragility fractures after age 40, falls in the past year, chronic diseases at baseline that may affect osteoporosis/fracture risk, tobacco use, enrolment in the Lilly patient support programme, and body mass index. Missing values for the propensity scores were imputed with the mean for continuous variables and the mode for categorical variables. In addition, PER analyses were used to assess vertebral and non-vertebral fragility fractures over 6-month intervals. Logistic regression adjusted for the covariates listed above as well as age, ethnicity, geographic region, and prior use of osteoporosis medications was used to assess odds of first fracture in a specific time period. Results are presented as hazard ratios (HR), odds ratios (OR), 95% confidence intervals (95% CI) and *p* values. Unless stated otherwise, all tests of statistical inference were conducted at a significance level of 0.05, and two-sided CIs were calculated at 95%.

Changes in EQ-5D-5L visual analogue scale (VAS) and back pain from baseline were analysed using mixed models for repeated measures (MMRM) with Kenward–Roger degrees of freedom and the compound symmetry covariance matrix. The MMRM was adjusted for the fixed effects of visit, teriparatide treatment (yes/no) and on treatment fracture (yes/no).

All analyses were conducted using SAS 9.4 (SAS Institute, Cary, NC, USA).

## Results

### Patient Disposition and Characteristics

**Fig. 1:**
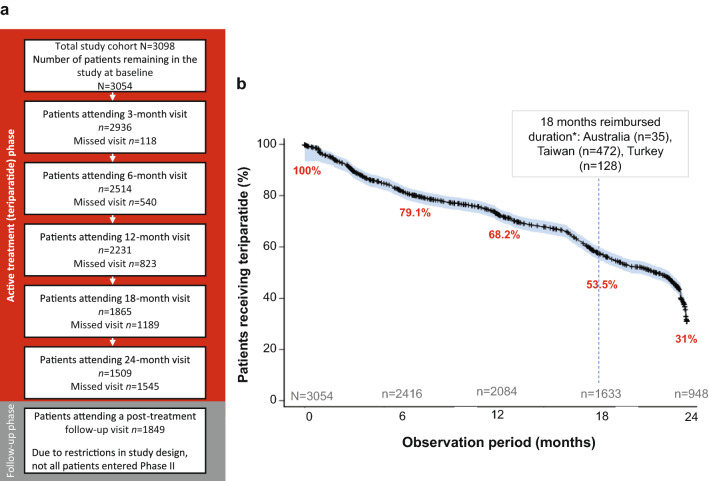
Study design flow (**a**) and teriparatide adherence (**b**). Panel **a** shows the study design flow, where *n* = the number of patients attending each follow-up visit during the treatment and post-treatment phases (independent of teriparatide treatment status). Percentages are based on number of patients initiating teriparatide treatment (*n* = 3054). Panel **b** demonstrates number and percentage of patients still taking teriparatide at each timepoint in the study, where *n* = the number of adherent patients at each time point during the study. *n = the number of participants enrolled at each site at baseline. Numbers of patients attending each visit (panel **a**) are not equal to numbers of patients still taking teriparatide (panel **b**) as numbers in panel **a** are irrespective of treatment status

A total of 3098 women were enrolled in the study and 3054 patients started teriparatide therapy (Fig. 1a). The study was conducted in the following countries: Argentina, Australia, Brazil, China, Colombia, Hong Kong, Israel, Kuwait, Lebanon, Malaysia, Mexico, New Zealand, Russian Federation, Saudi Arabia, Singapore, South Korea, Taiwan, Thailand, Turkey and United Arab Emirates. The final analysis presented includes the 3054 patients who initiated teriparatide treatment. The demographic and reproductive history of the study population have been previously reported [25] and here we present the updated baseline data for all 3098 patients after the final data lock (Table 1). The 44 patients that did not initiate teriparatide were not meaningfully different from those that initiated treatment.

**Table 1:** Patient baseline characteristics, medications, comorbidities, and reproductive history

Characteristics	All patients (*N* = 3098)
Age (years), mean (SD)	72.5 (10.4)
Race^a^, *n* (%)	
White	1645 (53.2)
Black/African American	37 (1.2)
Asian	1291 (41.8)
American Indian/Alaskan Native	92 (3.0)
Mixed race	24 (0.8)
Body mass index (kg/m^2^), mean (SD)	24.9 (4.5)
Bone mineral density (T-score), mean (SD)	
Lumbar spine	− 3.07 (1.39) (*n* = 1568)
Total hip	− 2.43 (1.11) (*n* = 715)
Femoral neck	− 2.59 (1.05) (*n* = 902)
Patients older than 40 years with ≥ 1 previous low trauma fracture, *n* (%)	1957 (63.2)
Patients older than 40 years with previous low trauma fracture (by number of fractures), *n* (%)	
No fractures	1140 (36.8)
1 fracture	1061 (34.3)
2 fractures	429 (13.9)
3 fractures	224 (7.2)
4 fractures	116 (3.7)
≥ 5 fractures	124 (4.0)
Patients with previous fractures according to the location of fractures, *n* (% of patients with fractures)	
Vertebral	1014 (51.8)
Nonvertebral	1013 (51.7)
Main nonvertebral^b^	771 (39.4)
Hip	440 (22.5)
Prior use of osteoporosis medication in the past 12 months, *n* (%) Maternal history of osteoporosis/hip fracture, *n* (%)	1738 (56.1) 512 (16.5)
Number of falls in previous year, mean (SD)	0.8 (1.2)
Hours active per week, mean (SD)	2.7 (5.5)
Medications^c^ *n* (%)	
Antihypertensives	825 (26.6)
Insulins/oral hyperglycemics	275 (8.9)
Thyroid hormones	192 (6.2)
Comorbidities^d^	
Hypertension	1302 (42.7)
Type 2 diabetes mellitus	384 (12.7)
Other chronic endocrine disease	285 (9.5)
Part of a Lilly patient support programme *n* (%)	1168 (37.7)
Reproductive history	
Age at onset of menopause (years), median (Q1, Q3)	50 (45.0, 52.0)
Number of fertile years^e^, median (Q1, Q3), years	35.0 (31.0, 38.0)
Parity^f^, *n* (%) patients	
0	239 (7.7)
1	268 (8.7)
2	527 (17.1)
3	603 (19.5)
4	460 (14.9)
≥ 5	991 (32.1)
Early menopause (age < 40 years), *n* (%)	145 (4.7)
Surgical menopause, *n* (%)	315 (11.2)

Percentages are calculated using the number of valid responses for each item as the denominator; this excludes any missing or unknown responses

*N* total number of patients available; *n* number of patients with valid (non-missing or unknown) values; *SD* standard deviation; *Q1* 1st quartile; *Q3* 3rd quartile

^a^Not answered (*n* = 8); Pacific islander (*n* = 1). Percentages are calculated using the number of valid responses as the denominator

^b^Radius, hip, humerus, tibia, pelvis and clavicle

^c^Medications related to osteoporosis risk taken by > 5% of all patients prior to baseline. Other medications included antidepressants (4.8% of all patients), anticoagulants/heparin (4.5%), glucocorticoids (4.0%), benzodiazepines (2.7%), antiarrhythmics (2.7%) and anticonvulsants (2.1%)

^d^The three most frequent comorbidities in the overall cohort are listed. Percentages are calculated using the number of valid responses as the denominator

^e^Age at menopause–age at menstruation

^f^Number of times given birth. Percentages are calculated using the number of valid responses as the denominator

The mean age in the overall cohort at baseline was 72.5 years. The majority of patients were either white or Asian and the mean body mass index for the overall cohort was 24.9 kg/m^2^. The median age at onset of menopause was 50 years with 145 (4.7%) patients having an early menopause (Table 1).

#### Fracture History and Bone Mineral Density

At baseline, 63.2% of patients reported at least one previous osteoporotic fracture, with 28.8% reporting at least two previous osteoporotic fractures. Mean bone mineral density *T* scores of patients with available values at baseline were − 3.07, − 2.43 and − 2.59 at the lumbar spine, hip and femoral neck, respectively. 51.8%, 51.7% and 22.5% of patients had one or more previous vertebral, non-vertebral (including hip fractures) or hip fractures, respectively (Table 1).

### Osteoporosis Treatment Adherence/Persistence

The number of patients that attended each visit and patient persistence with teriparatide are presented in Fig. 1a and b, respectively. For the 3054 patients who started teriparatide therapy, the median (95% CI) time to treatment discontinuation was 22.0 months (21.2, 22.8). The treatment persistence at 12, 18, and 24 months was 68.2%, 53.5%, and 31.0%, respectively (Fig. 1b) and at least 81% of patients had an MPR of at least 80% across the study period. A total of 211 patients reported treatment interruption. The mean (SD) sum of treatment interruptions was 165 days (160) with 207 (6.8%) patients reporting at least one treatment interruption/s of at least 28 days. After the active-treatment phase of the study, there was an optional post-treatment follow-up phase of up to 12 months, which was completed by 1849 (60.5%) patients (Fig. 1a).

During the study there were 1584 patients who discontinued teriparatide earlier than 24 months. Of those who discontinued, 31.4% was due to patient’s decision, 27.1% was due to economic reasons and 20.2% due to clinician’s decision. Additional reasons included AEs (6.4%, described below), lost to follow-up (3.9%), lack of efficacy (3.8%), death (2.1%) health system policy (1.1%), and others (4.0%).

### Fractures During Active Treatment Phase

During the 24-month active treatment period with teriparatide, 111 (3.6%) of the 3054 patients in the study, sustained a total of 126 clinical fragility fractures (2.98 fractures/100 patient-years). Of these 126 clinical fractures, 39 (31.0%) were clinical vertebral fractures and 87 (69.0%) were nonvertebral fractures including 24 (19.0%) hip fractures. Of the 111 patients who sustained a fracture during the 24 months, 97 patients sustained one fracture, 13 sustained two fractures, and 1 patient sustained three fractures.

**Fig. 2:**
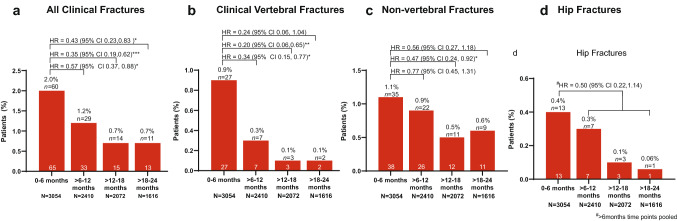
Number and percentage of patients with clinical fractures at each study timepoint. The number and percentage of participants sustaining a new clinical fragility fracture (**a**), clinical vertebral fracture (**b**), nonvertebral fractures (**c**), and hip fractures (**d**) at each timepoint during the study. The numbers at the top of each bar indicate the percentage and number (*n*) of patients that sustained at least one clinical fracture during each 6-month period. The total number of fractures sustained during each timepoint are listed at the bottom of each bar. The total number of patients that attended each follow-up visit is listed below each bar (N). The HR and 95% CI compare the rate of new clinical fragility fractures at each timepoint against the first 6-month period of teriparatide treatment (**a**–**c**). For hip fractures data from three time periods (> 6–12, > 12–18 and > 18–24 months) were pooled, due to the low numbers, and compared to the first 6-month period of treatment (**d**). *p < 0.05, **p < 0.01 and ***p < 0.001. As some patients sustained more than one fracture, the number of patients in (panel a) does not equal the sum of patients with clinical vertebral, non-vertebral, and hip fractures. *CI* confidence interval*, HR* hazard ratio

Figure 2a illustrates the number and percentage of patients with at least one clinical fracture in each 6-month period. The adjusted PER model with IPTW indicate that there was a significant decrease in the rate of new clinical fractures at each time point as compared to the first 6 months (Fig. 2a). Results indicate that the rate of new clinical fragility fractures significantly decreased during the > 6 to 12-month period, the > 12 to 18-month period and the > 18 to 24-month period as compared with the 0 to 6-month period (HR 0.57; 95% CI 0.37, 0.88; *p* = 0.012; HR 0.35; 95% CI 0.19, 0.62; *p* < 0.001; HR 0.43; 95% CI 0.23, 0.83; *p* = 0.011, respectively) (Fig. 2a).

Figure 2b–d show the number and percentage of patients with at least one clinical vertebral (Fig. 2b) non-vertebral (Fig. 2c) or hip (2d) fracture separately. Adjusted PER indicated that there was a statistically significant decrease in the rate of new clinical vertebral fractures during the > 6 to 12-month and > 12 to 18-month periods (HR 0.34; 95% CI 0.15, 0.77; *p* = 0.010; and HR 0.20; 95% CI 0.06, 0.65; *p* = 0.008, respectively) and in the rate of non-vertebral fractures during the > 12 to 18-month period only (HR 0.47; 95% CI 0.24, 0.92; *p* = 0.028) versus the 0 to 6-month period (Fig. 2b and c). Due to the small number of hip fractures sustained, data from the three timepoints were collapsed into one period (> 6–24 months). The hazard ratio of hip fractures was 0.5 although this finding did not reach statistical significance (95% CI 0.22, 1.14; *p* = 0.099).

Secondary, supportive analysis showed that the adjusted odds of first fracture significantly decreased during the > 6 to 12-month period, the > 12 to 18-month period and the > 18 to 24-month period (*p* = 0.032, 0.000, 0.004) when compared to the first 6 months of treatment, respectively (Table 2). The risk of first incident vertebral and nonvertebral fracture in relation to teriparatide treatment duration illustrates a similar trend and is detailed in Table 2.

**Table 2:** Risk of first incident clinical fracture in relation to time on treatment with teriparatide

Type of fracture	Observational period (months)	Number of patients^a^	Fracture rate per 100 patient-years	Patients with ≥ 1 fracture^b^, *n (%)*	Odds Ratio vs. 0–6 months (95% CI)^c,d^	*p* value^d^
All clinical fractures	0–6	3054	1.27	60 (2.0)	Reference group	
	> 6–12	2410	0.73	29 (1.2)	0.62 (0.39–0.96)	0.0321
	> 12–18	2072	0.44	14 (0.7)	0.35 (0.20–0.63)	0.0004
	> 18–24	1616	0.55	11 (0.7)	0.39 (0.20–0.73)	0.0036
Clinical vertebral fractures	0–6	3054	0.12	27 (0.9)	Reference group	
	> 6–12	2410	0.04	7 (0.3)	0.35 (0.15, 0.79)	0.0119
	> 12–18	2072	0.02	3 (0.1)	0.18 (0.05, 0.60)	0.0052
	> 18–24	1616	0.03	2 (0.1)	0.19 (0.04, 0.83)	0.0267
Clinical nonvertebral fractures	0–6	3054	1.83	35 (1.1)	Reference group	
	> 6–12	2410	1.40	22 (0.9)	0.78 (0.46, 1.33)	0.3665
	> 12–18	2072	0.85	11 (0.5)	0.45 (0.23, 0.89)	0.0209
	> 18–24	1616	1.03	9 (0.6)	0.49 (0.24, 1.02)	0.0568

*n* number of patients with valid (non-missing or unknown) values

^a^Number of participants with information regarding the number of sustained fractures during the observational period

^b^Some patients experienced fractures in more than one observational period

^c^Adjusted logistic regression model by age, body mass index, ethnicity, geographic region, tobacco use, prior use of osteoporosis medications or patient support programme, and history of fragility fractures after age 40, glucocorticoid-induced osteoporosis, diabetes (type I or II) or number of falls in the past year

^d^Compared with the 0 to 6-month period

### Health-Related Quality of Life

**Fig. 3:**
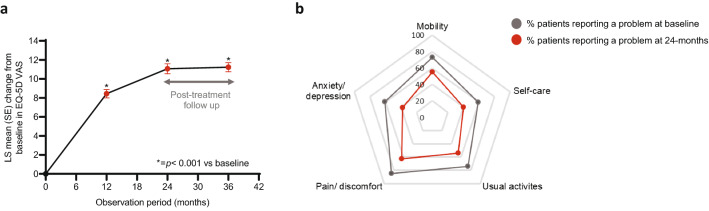
HRQoL. Change in EQ-5D-5L VAS score from baseline at 12 and 24 months post-treatment initiation and 36 months (12 months post treatment discontinuation) (**a**). Data are presented as LS mean change (SE) and analysed by MMRM; *p* < 0.001 for all time points compared to baseline. The unadjusted mean (SE) EQ-5D 5L VAS score at baseline, and at 12, 24, and 36 months was 61.1 (0.4), 69.6 (0.5), 72.2 (0.5), and 72.4 (0.5) respectively. **b** Radar chart of the proportion of patients reporting some or severe problems in the EQ-5D 5L dimensions at baseline or after 24 months of teriparatide treatment. Percentages were calculated based on the number of patients providing information at each time point. *EQ-5D-5L VAS,* EuroQol-5 dimension 5 level visual analogue scale; *LS* least squares; *MMRM* mixed model for repeated measures and *SE* standard error

The least squares (LS) mean (95% CI) EQ-5D-5L VAS was 61.1 (60.4, 61.9) at baseline. The LS mean change in EQ-5D-5L VAS from baseline showed statistically significant increases at all time points investigated during teriparatide treatment (12 and 24 months) compared to baseline (*p* < 0.001; Fig. 3a). This result was maintained after treatment discontinuation (up to 12 months of post-treatment follow-up; *p* < 0.001; Fig. 3a). Adjustment for patients with or without new fractures while on teriparatide treatment did not alter the statistical significance (Supplementary Figure S1a). Figure 3b shows the percentage of patients reporting problems in the five domains (mobility, self-care, usual activities, pain/discomfort, and anxiety/depression) of the EQ-5D-5L questionnaire at baseline and after 24 months of teriparatide treatment (Fig. 3b). Further information on the number of patients evaluable at baseline and 24 months post-treatment is provided in Supplementary Table S1.

Of the patients that reported problems with mobility, self-care, usual activities, pain/discomfort or anxiety/depression at baseline, 27%, 35%, 28%, 28%, and 45% reported no problems in the same area after 24 months of teriparatide treatment respectively (Supplementary Figure S2a). Of the patients that reported no problems with mobility, self-care, usual activities, pain/discomfort or anxiety/depression at baseline, 79%, 83%, 79%, 65%, and 84% still reported no problems in the same areas after 24 months of teriparatide treatment, respectively (Supplementary Figure S2b).

### Back Pain

**Fig. 4:**
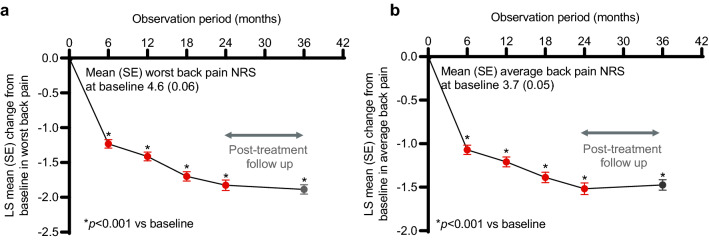
Back pain change during and after teriparatide treatment. Change in worst (**a**) and average (**b**) back pain score from baseline measured by the Back Pain NRS. Data are presented as LS mean change (SE) and analysed with MMRM; *p* < 0.001 for all timepoints compared to baseline. The unadjusted mean (SE) worst back pain at baseline and 6, 12, 18, 24, and 36 months was 4.6 (0.06), 3.4 (0.06), 3.2 (0.07), 2.9 (0.07), 2.8 (0.08), and 2.7 (0.07), respectively. The unadjusted mean (SE) average back pain at baseline, and 6, 12, 18, 24, and 36 months was 3.7 (0.05), 2.6 (0.05), 2.5 (0.06), 2.3 (0.06), 2.2 (0.07), and 2.2 (0.06), respectively. *LS* least squares, *MMRM* mixed model for repeated measures, *NRS* numeric rating scale and *SE* standard error

The LS mean (95% CI) worst and average back pain NRS at baseline was 4.6 (4.5, 4.7) and 3.7 (3.6, 3.8) respectively (Fig. 4a and b). There was a statistically significant decrease in the LS mean change from baseline in back pain NRS of both worst back pain and average back pain at each timepoint investigated during the treatment phase (6, 12, 18, and 24-months post-treatment initiation; Fig. 4a and b). This decrease was maintained during the 12-month post-treatment follow-up period for both worst and average back pain (Fig. 4a and b). Adjustment for patients with or without new fractures while on teriparatide treatment did not alter the statistical significance of these improvements (Supplementary Figure S1b and c).

### Post-teriparatide Cohort

Of the patients who were no longer taking teriparatide, 904 participants (30%) remained in the study for 0 to 3 months, 356 participants (12%) remained in the study for > 3 to 6 months, 884 participants (29%) remained on the study for > 6 to 12 months, 583 (19%) remained on the study for > 12 to 18 months, and 125 (4%) remained on the study for > 18 to 24 months. Of the patients with post-treatment data available, 82% took at least one osteoporosis medication after teriparatide discontinuation and 59% of patients took medication related to risk of osteoporosis. The most commonly taken osteoporosis drugs were denosumab (10.7%) and zoledronate (4.1%). In addition, 33% of patients took vitamin D and 31% took calcium supplement.

### Safety

Out of the 3054 patients who initiated teriparatide treatment, 181 (5.9%) patients experienced at least one AE (196 events), including 106 patients (3.5% of all patients) who experienced at least one SAE (117 events). The most frequently reported SAEs included hip fracture (12 patients [0.39%]; 13 events) and spinal compression fractures (9 patients [0.29%]; 12 events). A total of 65 (2.1%) patients are known to have died during the study.

## Discussion

This is the first study to show the effectiveness of teriparatide treatment in a real-world setting in postmenopausal women in Asia, Latin America, and the Middle East, regions with a growing burden of osteoporosis.

The results presented here indicate that postmenopausal women with osteoporosis who were at a high risk of fracture and were prescribed teriparatide, as part of standard clinical practice, had a significant reduction in the rate of fragility fractures after the first 6 months of treatment and reported an improvement in back pain and HRQoL. These results extend previous findings to Asian, Latin America, and Middle Eastern populations demonstrating similar effects of teriparatide to those shown in previous observational trials in other ethnicities [18–21]. ALAFOS included a cohort of patients from a broad geographic region. The patient populations across Asia, Latin America and the Middle East differ from populations studied in previous observational studies including differences in calcium and Vitamin D intake [16, 17, 27, 28]. Due to these differences, as well as differences in local clinical care, the baseline characteristics of the patients at risk for fractures and included in this study differed from those previously reported in other observational studies with teriparatide, particularly from those conducted in Europe: ExFOS and EFOS. The largest differences in the cohorts were prevalent fracture status and prior osteoporosis treatment [19, 20, 25, 31, 32]. In the current study, 36.8% of patients had not suffered an osteoporotic fracture before baseline. This ratio was smaller in the European studies: 14.6% in ExFOS and 8.1% in EFOS. Consequently, the percentage of participants who had received prior medication in the European studies was more than 85% as compared to 56.1% in the present study [19, 20, 22]. The anticipated lower fracture risk of the present population was reflected in the incidence of clinical fractures in the present study, 2% in the first 6 months as compared to 4.8% in EFOS, 3.1% in ExFOS and 3% in JFOS [19–21].

Overall, we report here that the rate of new clinical fragility fractures was significantly decreased during all time periods on treatment investigated (> 6–12, > 12–18, and > 18–24 months), as compared with the first 6 months of treatment. When clinical vertebral and non-vertebral fractures were considered separately, there was a decreasing trend at all time points. However, the reduction in rate of clinical vertebral fractures reached the level of statistical significance during the > 6 to 12-month and > 12 to 18-month periods only, whereas the rate of clinical non-vertebral fractures reached the level of statistical significance during the > 12 to 18-month period only. This may be due to the smaller sample size.

In general, these effectiveness results are consistent with previous observational studies from the USA, Europe, and Japan. In the USA, the DANCE observational study reported a decrease in non-vertebral fractures during all time points investigated [18]. In contrast, neither of the European observational studies (EFOS and ExFOS) reported a significant change in non-vertebral fractures during the active treatment time points. However, both studies did report a significant decrease in non-vertebral fractures during the post-treatment follow-up periods [19, 20]. These conflicting results could be explained by the fact that DANCE had a larger sample size and a population with less advanced disease when compared with patients in the European studies and is thus more similar to the present study population.

In terms of vertebral fractures, the results from EFOS show a decrease in the adjusted odds of clinical vertebral fractures during the last 6 months of teriparatide treatment and during the post-treatment follow-up periods [19]. Similarly, the results from ExFOS show a decrease in the adjusted odds of clinical vertebral fracture during both the teriparatide treatment phase and the post-treatment phase [20].

In this study, results showed fewer fractures during each 6 month period compared to the reference first 6 months with statistical significance observed for most intervals.

ALAFOS had a shorter median time to treatment discontinuation than both ExFOS and DANCE (22.0 months vs. 23.6 months and 23 months, respectively). This shorter duration could be, in part, due to differences in prescribing patterns and reimbursement policy. Indeed, a lack of reimbursement after 18 months of treatment affected 635 patients from three countries (Fig. 1). In addition, the small number of incident fractures observed, likely due to the lower absolute fracture risk in the ALAFOS study cohort, may result in a lack of power to detect a significant change at the later time point during the study. Moreover, due to the drop-out rate, the results presented here need to be interpreted with this limitation in mind. In an attempt to account for the drop-out rate, fracture rates were modelled using PER with IPTW. This analysis attempts to match patients across each period so that ‘similar patients’ (based on variables used in the propensity score to generate the weights) are being compared across the periods.

Previous observational studies have not reported a significant reduction in hip fractures, and our results are in line with these previous findings. However, integrated analysis of four observational studies, including 8828 patients, demonstrated that teriparatide treatment is associated with a statistically significant decrease in the hip fracture rate [17]. The hip fracture results presented here follow a similar trend. Although there was no statistically significant reduction observed with the adjusted hazard ratio, this may be due to the low incidence of hip fractures and the limited sample size in this study.

The negative effect of osteoporosis on quality of life has been well reported previously, as has the reduction in back pain and increase in HRQoL of osteoporosis patients taking teriparatide. As such, this study also assessed the effect of teriparatide on HRQoL in a real-world setting in the ALAFOS regions. Patients reported an improvement in back pain and in HRQoL, an improvement which remained significant after adjusting for patients with no new fractures.

Yu et al*.*, have reported that the risk of any clinical, vertebral, and non-vertebral fractures decreases as teriparatide adherence and persistence increase [33]. Most patients in ALAFOS (≥ 81%) maintained a high level of adherence (≥ 80%) during the active treatment phase. The commonly recommended treatment duration for teriparatide is 24 months [34]. However, the treatment persistence observed in this study was below the recommended 2 years. We report here that only 31% of patients were still taking teriparatide at 24 months. Although this is lower than a previous observational study in Japan [21], it is similar to a recent meta-analysis reporting that the median teriparatide treatment persistence was 29.5% at 24 months across eight studies [35].

A recent meta-analysis has shown that treatment adherence and persistence rates are suboptimal among patients with osteoporosis in general [35]. Furthermore, an integrated study by Silverman et al*.,* reported that teriparatide treatment for more than 18 months confers additional benefit for reducing hip fractures as compared to shorter durations of teriparatide therapy [17]. Together, this suggests that medical strategies need to be developed to improve treatment adherence and thus the benefit to patients, especially in the regions included in this study. Sato et al*.,* have highlighted the value of patient support programmes for treatment adherence and persistence with daily teriparatide [36]. Of the patients that initiated teriparatide (*N* = 3054), 1159 (38%) patients received support from patient support programme and 1895 (62%) patients were not part of a patient support programme.

In terms of safety, the percentage of patients experiencing at least one AE or an SAE was lower than in previous studies [18, 20, 21] and there were no new safety signals or reports of osteosarcoma during this study.

One of the benefits of this study is that ALAFOS has a broader generalizability compared to RCTs. ALAFOS included a large sample size of a diverse range of patients. The lack of strict inclusion and exclusion criteria, used in clinical trials, make the results presented here applicable to the general population. Patients participating in the study were receiving concomitant medications and had a number of comorbidities including hypertension and Type 2 Diabetes, reflecting routine clinical practice in real-world settings (Table 1). With the growing burden of risk of fracture globally, and especially in the regions included in this study [2], real-world evidence of treatment effectiveness is essential to complement RCT efficacy data. Although teriparatide treatment patterns might differ between countries, all real-world studies on teriparatide effectiveness in populations covering Latin America, Asia–Pacific, Europe, the USA, and Japan provided similar results confirming that daily treatment reduces the risk of clinical vertebral and non-vertebral fractures.

## Limitations

As with all observational studies, the single-armed design and thus lack of a comparator, must be taken into consideration when interpreting the results. Another limitation, related to the real-world design of this study, was that X-ray assessment of asymptomatic vertebral fractures was not systematically performed since several other ways of fracture validation were also accepted and commonplace in the health-systems encountered. The results must also be interpreted with caution due to selection bias and unmeasured confounding, however, of note, results from this study mirror what has been observed in clinical trials. Given the observational design, data on self-reported outcomes were not available for all patients and all follow-up time points and, also, treatment end dates were missing in some cases. Unmeasured bias can also be due to drop-out, which is frequent in observational real-world studies. Due to restrictions in the study design not all patients were able to enter the post-treatment follow-up. In this study, 1205 patients (39.5%) were not observed after treatment discontinuation. Furthermore, the patients who did enter the post-treatment phase were observed for varying durations and there was a large variation in the duration of post-treatment follow-up. It is possible that patients stopped taking teriparatide or were lost to follow up due to their fracture status. However, although less than half of patients were seen at the 24-month visit, time to event analysis was used to account for patients who left the study, died, or were lost to follow up. Furthermore, using the PER with IPTW attempts to match similar patients in different time periods, thus attempting to reduce some of the potential bias.

## Conclusion

Postmenopausal women with osteoporosis who were at high risk of fracture had a significant reduction in the rate of fragility fractures after the first 6 months of teriparatide treatment and concurrent improvements in back pain and HRQoL. The results described here provide data on the real-world effectiveness of teriparatide for treating osteoporotic patients in the ALAFOS regions and are consistent with other clinical and observational studies showing reduction of fractures after 6 months of teriparatide treatment.

## Supplementary Information

Below is the link to the electronic supplementary material.

Supplementary file1 (DOCX 430 kb)

## Data Availability

Lilly provides access to all individual participant data collected during the trial, after anonymization, with the exception of pharmacokinetic or genetic data. Data are available to request in a timely fashion after the indication studied has been approved in the US and EU and after primary publication acceptance. No expiration date of data requests is currently set once they are made available. Access is provided after a proposal has been approved by an independent review committee identified for this purpose and after receipt of a signed data sharing agreement. Data and documents, including the study protocol, statistical analysis plan, clinical study report, blank or annotated case report forms, will be provided in a secure data sharing environment for up to 2 years per proposal. For details on submitting a request, see the instructions provided at www.clinicalstudydatarequest.com.
