# Supplementary material for: The Effect of Teriparatide Treatment on the Risk of Fragility Fractures in Postmenopausal Women with Osteoporosis: Results from the Asian and Latin America Fracture Observational Study (ALAFOS)
Source: Calcif Tissue Int. 2021 Aug 20;110(1):74–86. doi: 10.1007/s00223-021-00895-4 (PMC8732800; doi:10.1007/s00223-021-00895-4)
Supplement: Supplementary file 1 — Supplementary file1 (DOCX 430 kb) [file 223_2021_895_MOESM1_ESM.docx]

**Supplementary materials**

**The Effect of Teriparatide Treatment on the Risk of Fragility Fractures in Postmenopausal Women with Osteoporosis: Results from the Asian and Latin America Fracture Observational Study (ALAFOS)**

1. Supplementary methods
   - Sample size
2. Supplementary results

- Figure S1: Adjusted mean HRQol and back pain change from baseline
- Table S1: EQ-5D-5L at baseline and after 24 months of teriparatide treatment
- Figure S2: Change in EQ-5D-5L from baseline to 24-months post-teriparatide treatment

1. **Supplementary methods**

**Sample size**

To achieve approximately 80% power, the study enrolled approximately 3000 patients in total. At the time of the study design, there was limited information regarding time to clinical fracture in relation to adherence in the ALAFOS regions to inform the sample size calculation. The sample size calculation was based on the following assumptions and the simulation of time-to-first fracture data:

1. Two-sided statistical significance of 0.05

2. At least 80% power to study the fracture risk reduction in patients with longer time on therapy compared with patients with shorter time on therapy

3. The estimated effect size of about 44% relative risk reduction in patients with longer time on therapy compared with patients with shorter time on therapy

4. The nonvertebral fracture rate in the DANCE study was 3.2 per 100 patient-years for 0 to 6 months on therapy and 1.8 for 6 to 24 months on therapy. Therefore, for the first 0 to 6 months on therapy the time-to-fracture had an exponential distribution with parameter approximately 0.032, and for >6 to 24 months on therapy the time-to-fracture had an exponential distribution with parameter approximately 0.018

5. Data simulation (ie, generate time-to-fracture for all patients) using the piecewise exponential distribution with the specified parameters and right censoring at 24 months

6. Power was computed by analyzing the simulated fracture time data using PROC LIFEREG in Statistical Analysis System (SAS). The life regression model has time period (either beginning [0 to 6 months] or later [6 to 24 months]) as the class variable. The simulation was repeated 1000 times to compute the power: power = (number of times p-value for period effect ≤.05)/1000

7. Patients who drop out of the study were incorporated using an exponential distribution, with an annual dropout rate of approximately 22.5%, resulting in approximately 40% dropouts over 24 months

**2. Supplementary results**

**
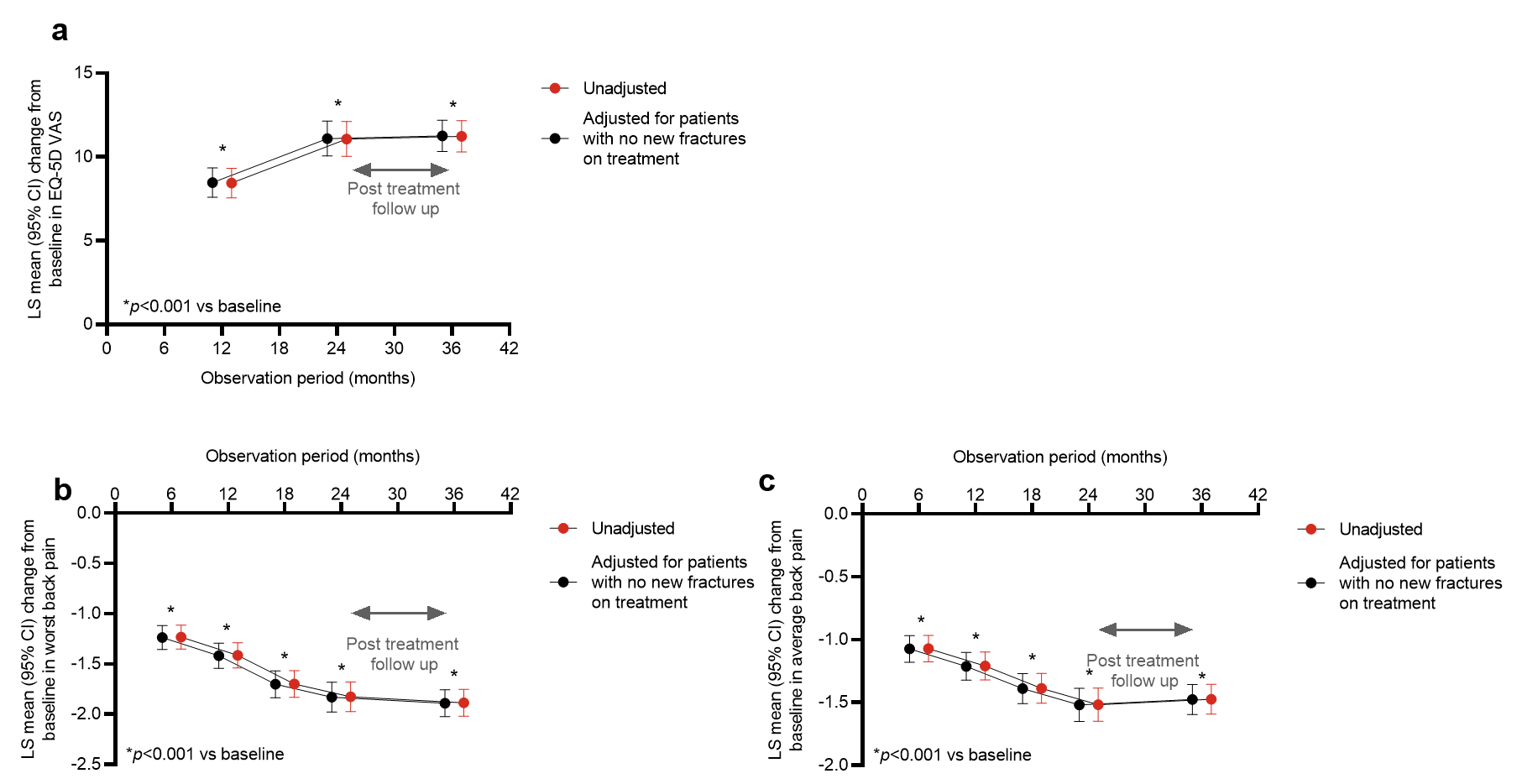
**

**Supplementary Figure S1 Adjusted mean HRQol and back pain change from baseline**. Change in EQ-5D-5L VAS score (a), worst back pain (b), and average back pain (c) from baseline. Back pain was measured by the Back Pain NRS Data are presented as LS mean change (95% CI) and analysed with MMRM adjusted for teriparatide treatment (yes/no) and on treatment fracture (yes/no). **p*<0.001 vs baseline at all time points for adjusted and unadjusted values. *EQ-5D VAS,* EuroQol-5 dimension 5L visual analogue scale, *LS* least squares *MMRM* mixed model for repeated measures, *NRS* numeric rating scale and *CI* confidence interval.

**Supplementary Table S1** EQ-5D-5L at baseline and after 24 months of teriparatide treatment

| **EQ-5D dimension** |  | **Baseline** | **24 months of teriparatide** |
| --- | --- | --- | --- |
| **Mobility** *N* |  | 2963 | 1294 |
| Patients (*n* [%]) showing | No problems | 802 (27) | 582 (45) |
|  | Slight problems | 2161 (73) | 712 (55) |
|  | *Missing* | *134* | *1803* |
| **Self-care** *N* |  | 2965 | 1294 |
| Patients (*n* [%]) showing | No problems | 1228 (41) | 775 (60) |
|  | Slight problems | 1737 (59) | 519 (40) |
|  | *Missing* | *132* | *1803* |
| **Usual activities** *N* |  | 2963 | 1293 |
| Patients (*n* [%]) showing | No problems | 772 (26) | 594 (46) |
|  | Slight problems | 2191 (74) | 699 (54) |
|  | *Missing* | *134* | *1804* |
| **Pain/ discomfort** *N* |  | 2943 | 1238 |
| Patients *n* [%]) showing | No problems | 435 (15) | 455 (37) |
|  | Slight problems | 2508 (85) | 783 (63) |
|  | *Missing* | *154* | *1859* |
| **Anxiety/ depression** *N* |  | 2900 | 1278 |
| Patients (*n* [%]) showing | No problems | 1124 (39) | 796 (62) |
|  | Slight problems | 1776 (61) | 482 (38) |
|  | *Missing* | *197* | *1819* |

EQ-5D, EuroQol-5 dimension, Missing, patients not reporting data at this timepoint. N, number of evaluable patients at each time point (denominator for the percentages); *n*, number of patients reporting that characteristic.

**
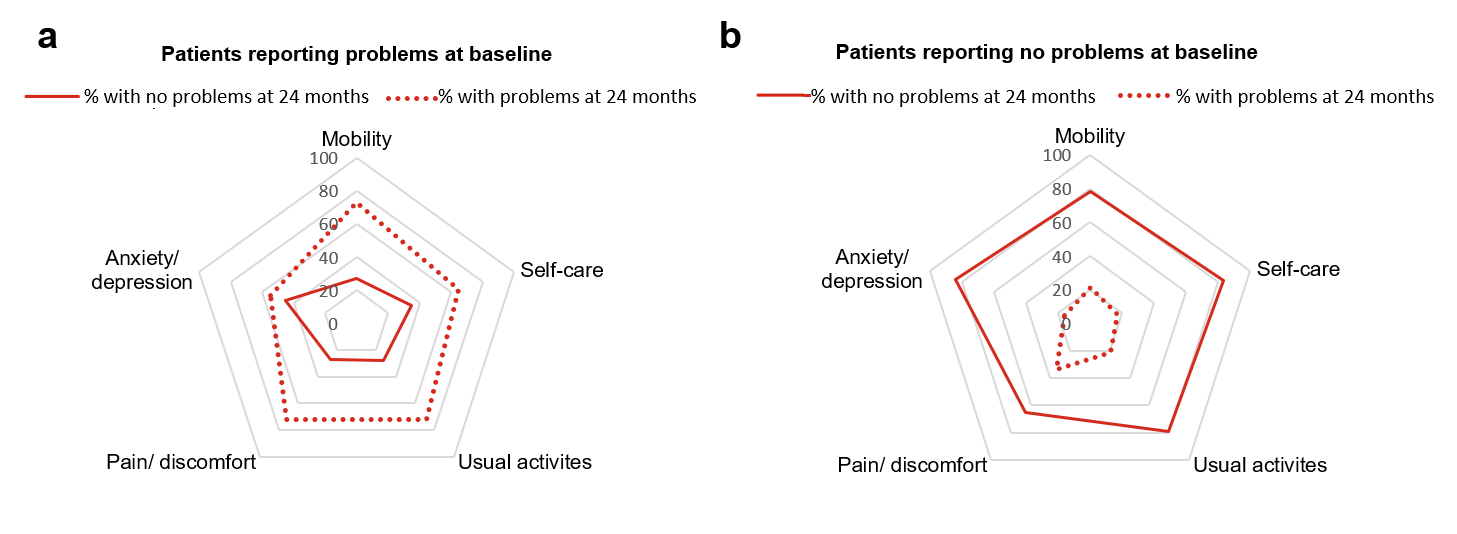
**

**Supplementary Figure S2 Change in EQ-5D-5L from baseline to 24-months post-teriparatide initiation.** Radar chart of the proportion of patients reporting problems at baseline and no problems after 24 months of teriparatide treatment (a) and the proportion of patients reporting no problem at baseline and a problem after 24 months of teriparatide treatment (b) in the EQ-5D-5L dimensions. Percentages were calculated based on the number of patients providing information at each time point. *EQ-5D-5L* EuroQol-5 dimension 5L.
